# Characterization of the MUC1-C Cytoplasmic Domain as a Cancer Target

**DOI:** 10.1371/journal.pone.0135156

**Published:** 2015-08-12

**Authors:** Deepak Raina, Praveen Agarwal, James Lee, Ajit Bharti, C. James McKnight, Pankaj Sharma, Surender Kharbanda, Donald Kufe

**Affiliations:** 1 Dana-Farber Cancer Institute, Harvard Medical School, Boston, MA, 02215, United States of America; 2 Genus Oncology, Boston, MA, 02118, United States of America; 3 LeadInvent, New Delhi, India; 4 Boston University School of Medicine, Department of Medicine, Boston, MA, 02118, United States of America; 5 Boston University School of Medicine, Department of Physiology & Biophysics, Boston, MA, 02118, United States of America; IISER-TVM, INDIA

## Abstract

Mucin 1 (MUC1) is a heterodimeric protein that is aberrantly expressed in diverse human carcinomas and certain hematologic malignancies. The oncogenic MUC1 transmembrane C-terminal subunit (MUC1-C) functions in part by transducing growth and survival signals from cell surface receptors. However, little is known about the structure of the MUC1-C cytoplasmic domain as a potential drug target. Using methods for structural predictions, our results indicate that a highly conserved CQCRRK sequence, which is adjacent to the cell membrane, forms a small pocket that exposes the two cysteine residues for forming disulfide bonds. By contrast, the remainder of the MUC1-C cytoplasmic domain has no apparent structure, consistent with an intrinsically disordered protein. Our studies thus focused on targeting the MUC1 CQCRRK region. The results show that L- and D-amino acid CQCRRK-containing peptides bind directly to the CQC motif. We further show that the D-amino acid peptide, designated GO-203, blocks homodimerization of the MUC1-C cytoplasmic domain in vitro and in transfected cells. Moreover, GO-203 binds directly to endogenous MUC1-C in breast and lung cancer cells. Colocalization studies further demonstrate that GO-203 predominantly binds to MUC1-C at the cell membrane. These findings support the further development of agents that target the MUC1-C cytoplasmic domain CQC motif and thereby MUC1-C function in cancer cells.

## Introduction

Epithelia are layers of laterally connected cells with apical-basal polarity that are protected from the external environment by a mucous barrier [1]. The mucin family of secreted and transmembrane glycoproteins evolved in metazoans to afford protection of epithelia that, for example, line (i) the respiratory and gastrointestinal tracts, and (ii) ducts in specialized organs [1]. Mucin 1 (MUC1) is a transmembrane member of this family that was identified by its overexpression in human breast cancers [2]. MUC1 consists of two subunits that derive from autocleavage of a single polypeptide and, in turn, form a heterodimeric complex at the apical membrane of normal epithelial cells [1]. The MUC1 N-terminal subunit (MUC1-N) contains tandem 20-amino acid (aa) repeats that are modified by *O*-glycans. The MUC1 C-terminal subunit (MUC1-C) is the transmembrane component of the heterodimer and anchors MUC1-N to the cell surface. MUC1 is overexpressed in diverse carcinomas, supporting the notion that upregulation of the MUC1-N/MUC1-C complex represents a subversion of its normal function to promote the survival of cancer cells [1]. In this context and in addition to participating in the mucous barrier, glycosylated MUC1-N may enhance cell surface receptor function to support growth and survival [3]. Moreover and in association with loss of polarity, MUC1-C interacts with cell surface molecules, such as receptor tyrosine kinases, and promotes their activation and downstream signaling [4]. Significantly, overexpression of MUC1-C is sufficient to confer anchorage-independent growth and tumorigenicity, supporting the oncogenic function of this subunit [5].

MUC1-C consists of a 58-aa extracellular domain, a 28-aa transmembrane region and a 72-aa cytoplasmic domain. The MUC1-C extracellular domain includes an NPG motif with the asparagine residue subject to *N*-glycosylation [6]. Binding of galectin-3 to that glycosylated NPG site promotes the formation of extracellular bridges between MUC1-C and other cell surface molecules, such as the epidermal growth factor receptor (EGFR) [6]. The MUC1-C cytoplasmic domain (MUC1-CD) contains a CQC motif adjacent to the transmembrane region, which is necessary and sufficient for the formation of MUC1-C homodimers [7,8]. Importantly, mutation of the CQC motif to AQA blocks import of MUC1-C to the nucleus and mitochondrial outer membrane [7,9,10]. Moreover, the CQC→AQA mutation abrogates the MUC1-C oncogenic function, supporting the importance of MUC1-C homodimerization in driving intracellular signals that confer growth and survival [7,11]. Accordingly, the MUC1-C CQC motif has emerged as an attractive target for the development of peptide and small molecule inhibitors that block MUC1-C homodimerization and function [12,13].

The present studies have investigated the structure of the MUC1-C cytoplasmic domain based on its potential importance as a cancer target. We demonstrate that the MUC1-C cytoplasmic CQC motif resides in a druggable configuration and that the remainder of the cytoplasmic domain is unstructured, consistent with other oncogenic molecules that direct the activation of multiple signaling pathways [14]. We also show that the MUC1-C cytoplasmic domain can be selectively targeted with peptides that directly bind to the CQC motif and block MUC1-C homodimerization in vitro and in cells.

## Materials and Methods

### Molecular Dynamics

MUC1-CD (amino acids 1–15) structure was built *ab-inito* with the preferred phi/psi angles of the respective amino acids. The resultant atomic structure with explicit water molecules and ions was studied without a membrane bi-layer using Assisted Model Building with Energy Refinement (AMBER) [15]. Following stabilization steps of minimization, heating and equilibration, short 15 ns simulations were conducted to generate 1500 conformations separated by 10 picoseconds, which were further analyzed for Root Mean Square Deviation (RMSD). In additional studies, the MUC1-C membrane region, which has a predominant helix conformation as predicted by THMHMM and TMPred, was inserted in a pre-equalibrated 1-palmitoyl-2-oleoyl-sn-glycero-3-phosphocholine (POPC) bi-layer and solvated with explicit water and ions. The remaining cytoplasmic domain amino acids were built with their preferred phi/psi angles. Nanoscale molecular dynamics (NAMD) [16] was used to perform the molecular-dynamics study.

### NMR Analysis of MUC1-CD

His-MUC1-CD was expressed from the pET-MUC1-CD plasmid (Novagen, Billerica, MA) and the ^15^N-labeled protein was prepared as described [17]. The NMR sample consisted of 1.7 mg/ml of His-MUC1-CD, 1% D_2_O, reference compound TMSP (250 μM) and 10 mM d_10_-DTT. The pH was adjusted to 5.0 with the addition of 1 M HCl. One and two dimensional ^15^N heteronuclear single quantum coherence (HSQC) data collections were performed at 20°C and 10°C on a 500 MHz Bruker Advance III NMR system at the Boston University Core facility for structural NMR. Data analysis was generated using Bruker’s topspin software.

### Measurement of Binding Affinities

Binding affinities of FITC-GO-202 and FITC-GO-203 were determined by the saturation binding method [18]. In short, 96-well glutathione- or nickel-coated plates (Thermo Fisher, Waltham, MA) were incubated with 150 μl of PBS buffer containing 21.45 μg/ml of GST-MUC1-CD, GST-MUC1-CD(AQA) or His-MUC1-CD overnight at 4°C followed by washing with 50 mM Tris and 150 mM NaCl containing 0.1% Tween-20 (TBST), and blocking with 0.5% BSA in TBS. The plates were then washed 3 times with TBST. FITC-GO-202 or FITC-GO-203 was added to the wells at 0.19 μM to 25 μM. Nonspecific binding was determined in the presence of GST alone. Plates were incubated for 1 h at 37°C on a shaker followed by washing 3 times with binding buffer. Fluorescence intensity was determined on an Infinite M1000 PRO Tecan Microplate Reader (Tecan, NC) with excitation at 490 nm and emission at 525 nm. GraphPad Prism 4.0 Software (San Diego, CA) was used to calculate the equilibrium dissociation constant (Kd) by a non-linear regression method.

### Complex Characterization by MALDI-TOF-MS

Binding experiments with MUC1-CD and GO-203 were performed as described [19]. Complexes were eluted from the glutathione beads, dialyzed and concentrated using amicon ultra centrifugal filters (Millipore, Billerica, MA). The complexes were then subjected to non-reducing electrophoresis and stained with Coomassie Blue. Protein bands were excised and analyzed by MALDI-TOF-MS as described [20]. Briefly, gels were cut into small, uniform pieces; the gel pieces were dehydrated with acetonitrile and then rehydrated in 100 mM ammonium bicarbonate followed by 2 washing and drying cycles with ammonium bicarbonate and acetonitrile, respectively. After complete dehydration, gel pieces were suspended in 12.5 ng/μl trypsin in 50 mM ammonium bicarbonate (50 μl). In-gel digestion was carried out at 37°C for 10–12 h and the samples were acidified with 50 μl of 0.5% TFA. Thirty μl of this mixture was desalted by C-18 containing Zip-Tip (Millipore, Billerica, MA). Trypsin-digested peptides were analyzed by MALDI-TOF-MS (ABI-4800). MALDI-TOF-MS analyses were performed using a Reflector method that was optimized for acquisition for the mass range of 700–3500 amu. Data was collected and analyzed using the data Explorer software package (AB-Sciex, Framingham, MA) and MASCOT (ver.2.0.04, Matrix Science, Boston, MA). MS-bridge analysis was done using Protein Prospector (ver.4.27.2 basic and ver.5.0, UCSF). The database search parameters were: (i) tryptic digest, (ii) oxidized Met, (iii) 2 missed cleavages, and (iv) disulfide bridge with maximum link molecules set to 2 and a mass tolerance of 50 ppm. Searching was conducted against GST-MUC1-CD and GO-203.

### Plasmid Construction

Vectors expressing GST-MUC1-CD, GST-MUC1-CD(AQA) and His-MUC1-CD, GFP-MUC1-CD and FLAG-MUC1-CD have been described [7]. His-tagged MUC1-CD (rat and dog) were cloned in pET28a vector (Clontech, Mountain View, CA) and expressed in *E*. *coli* BL21(DE3).

### *In Vitro* Dimerization

Purified His-MUC1-CD was incubated in PBS or increasing amounts of GO-203 for 1 h at room temperature as described [19]. Proteins were then separated in non-reducing polyacrylamide gels and analyzed by immunoblotting.

### Cell Culture

Human ZR-75-1 breast cancer (ATTC) and H1975 lung cancer (ATTC) cell lines were grown in RPMI 1640 supplemented with 10% heat-inactivated fetal bovine serum (HI-FBS), 100 units/ml penicillin, and 100 μg/ml streptomycin. 293T cells were grown in DMEM with 10% HI-FBS, antibiotics, and 2 mmol/L L-glutamine. Cells were treated with FITC conjugated-GO-202 (FITC-GO-202), FITC-GO-203 or unconjugated GO-203 synthesized by the MIT Biopolymer Laboratory (Cambridge, MA) and AnaSpec, Inc (San Jose, CA). The p3XFLAG-CMV-10 (Sigma, St. Louis, MO), pEGFP-C1 and pDsRed-Monomer-N1 (Clontech, Mountain View, CA) vectors were used for transfection of rat and dog MUC1-CD and full length human MUC1 in 293T cells in the presence of Lipofectamine 2000 (Life Technologies, Grand Island, NY).

### Immunoprecipitation and Immunoblot Analysis

Cell lysates were prepared as described [19]. Soluble proteins were immunoprecipitated with anti-FLAG (Sigma, St. Louis, MO), anti-MUC1-C (Thermo Fisher Scientific, Waltham, MA) or a control IgG. The precipitates and lysates not subjected to immunoprecipitation were immunoblotted with anti-MUC1-C, anti-GFP (Abcam, Cambridge, MA), anti-FLAG and anti-FITC (Life Technologies, Grand Island, NY). Reactivity was detected with horseradish peroxidase-conjugated secondary antibodies and chemiluminescence.

### Analysis of GO-203 Localization

293T cells were transfected with MUC1-pDsRed-Monomer-N1 in the presence of Lipofectamine 2000 for 48 h. The cells were then incubated with FITC-GO-203 for 3 h and examined using a Nikon-deconvolution wide-field epifluorescence system with a 100x oil immersion objective. Images were captured using NIS-element software (Nikon) and analyzed by ImageJ software.

## Results

### Analysis of MUC1-C Cytoplasmic Domain Structure

To define the structure of the 72-amino acid MUC1 cytoplasmic domain (MUC1-CD), we first used multiple conditions to generate crystals with purified MUC1-CD protein. These attempts at crystallization proved unsuccessful, prompting us to explore molecular simulation studies. We first turned to homology modeling as one approach to map the structural aspects of MUC1-CD. Notably, however, MUC1-CD exhibited little if any sequence homology with known proteins. Accordingly, we studied secondary structure predictions for MUC1-CD using different methods, including ROBETTA [21] and IGB-SSPro [22]. The results of these studies collectively indicated that MUC1-CD does not conform to a typical secondary structure.

**Fig 1:**
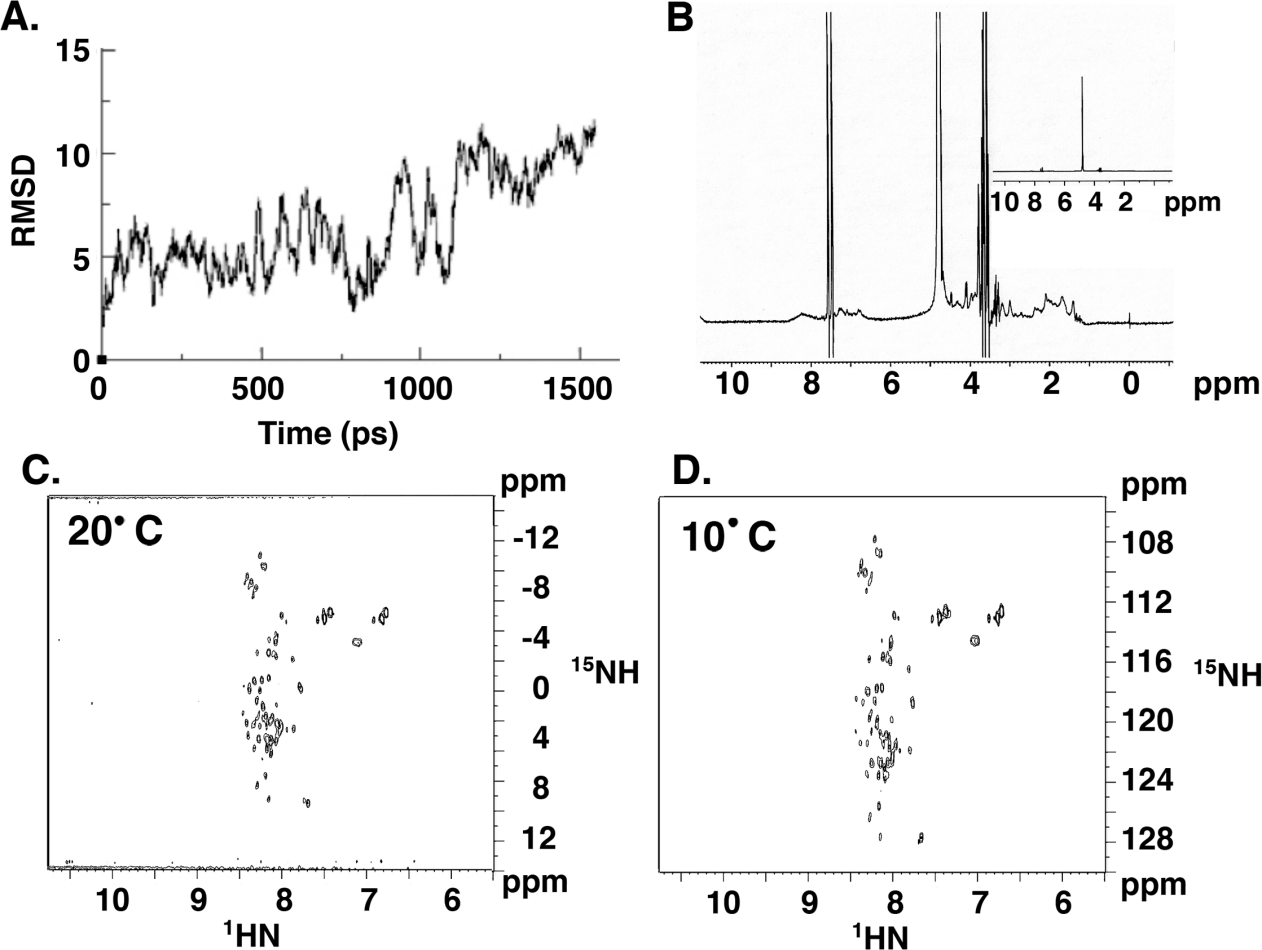
RMSD analysis of MUC1-CD (1–15). (A) RMSD of snapshots obtained for the initial *ab-initio* structure of MUC1-CD(1–15). Y-axis is the RMSD and X-axis is the snapshot sequence (picoseconds). (B) Proton NMR spectra (1D) and ^15^N-HSQC spectra of MUC1-CD(1–15) were acquired at 500 MHz at (C) 20°C and (D) 10°C at pH 5.0.

We thus focused our efforts on the first 15 amino acids of MUC1-CD (CQCRRKNYGQLDFIP) by studying its atomistic molecular dynamics. The MUC1-CD(1–15) model was built *ab-inito* with the preferred phi/psi angles of amino acids. The resulting structure was then studied with explicit water and ions. Using short 15 nanosecond simulations (1500 conformations separated by 10 picoseconds) with AMBER and as demonstrated by RMSD, we found that the MUC1-CD(1–15) sequence is also inherently unstructured with no preferred conformations (Fig 1A). In addition, NMR analysis of MUC1-CD confirmed the absence of a detectable secondary structure (Fig 1B–1D), as shown by the presence of the peaks between 7.5 and 8.5 ppm, consistent with MUC1-CD being an unstructured protein.

**Fig 2:**
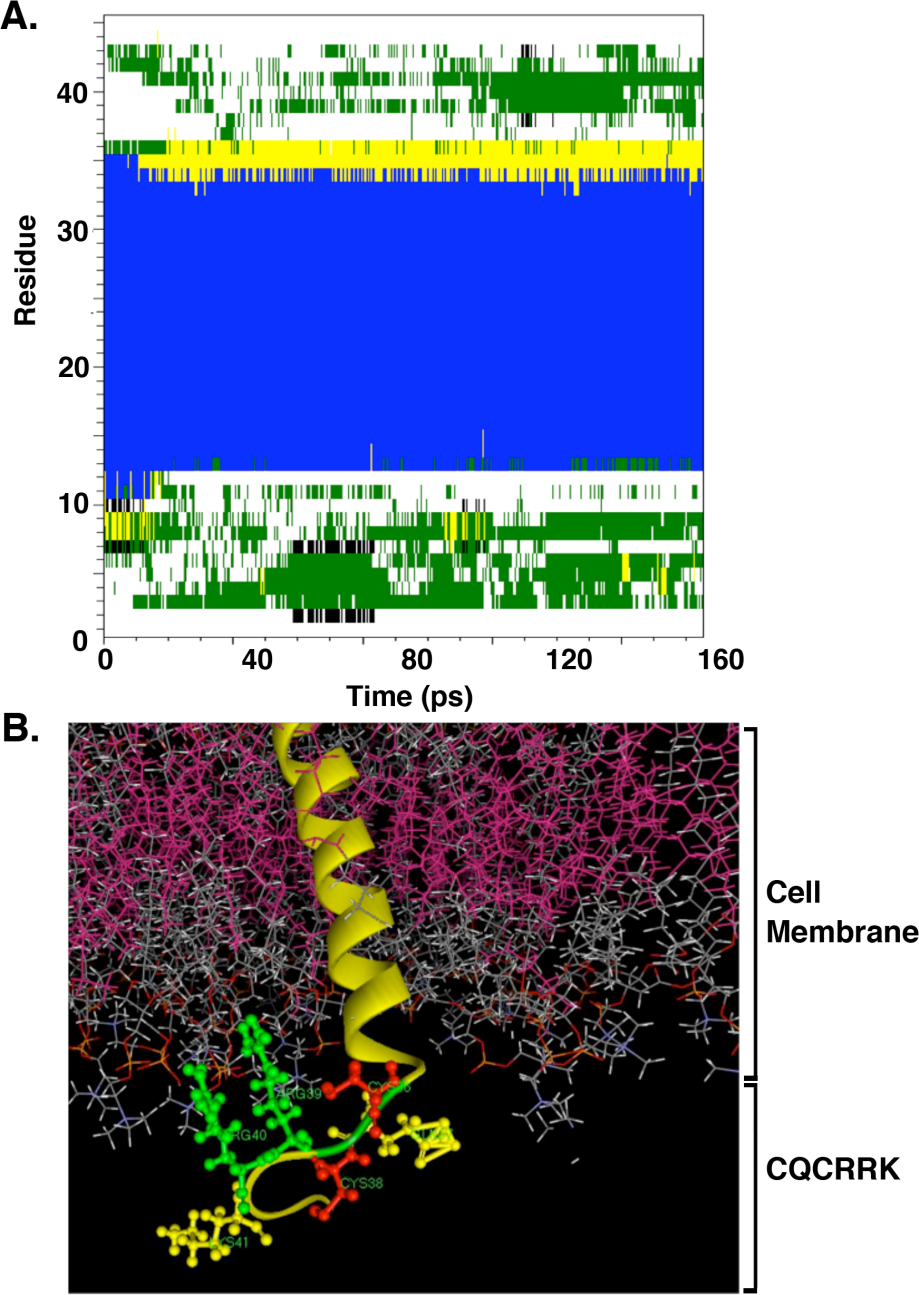
Secondary structure propensity analysis. (A) Secondary structure distribution of snapshots were obtained with respect to simulated time and assessed with the DSSP algorithm. Y-axis is the residue sequence: (1 to 7—SAQSGAG), (8 to 35 –VPGWGIALLVLVCVLVALAIVYLIALAV), (36 to 45 –CQCRRKNYGQ). X-axis is time in picoseconds (ps). (B) The figure depicts a region of the lipid bilayer highlighted as pink, red and orange sticks representing phospholipid monomers with the helical MUC1-C TMD. The cytoplasmic residues of MUC1-C consisting of CQCRRK are highlighted as a ball and stick model. The positively charged arginines are pulled up and locked towards the negatively charged phosphate groups.

MUC1-CD(1–15) contains a CQC motif followed by three positively charged residues (RRK). The cytoplasmic CQCRRK region is located adjacent to the cell membrane (predicted by TMPred & Phobius) and thereby could play a role in MUC1-CD structure due to charge-charge interactions with the negatively charged lipid layers. We therefore studied the potential effect of the cell membrane lipid layer on the CQCRRK residues. A 45 amino acid MUC1 sequence (SAQSGAG-VPGWGIALLVLVCVLVALAIVYLIALAV-CQCRRKNYGQ) was built *ab-initio* with part of the MUC1-C/extracellular domain (MUC1-C/ECD; SAQSGAG), the 28-amino acid MUC1-C/transmembrane domain (MUC1-C/TMD; VPGWGIALLVLVCVLVALAIVYLIALAV) and the first 10 residues of MUC1-CD (CQCRRKNYGQ). As determined by molecular dynamics studies, the TMD has a predominant helix conformation (Fig 2A). The remaining amino acids were built with their preferred phi/psi angles. The resulting structure was inserted in a representative membrane patch (POPC bilayer) and atomistic molecular dynamics studies were performed for 55 ns using NAMD software. We observed an equilibrated structure with stable RMSD and a preferred conformation (turn) at the CQC region (Fig 2A). We also found that the two Arg residues preferably orient towards the membrane because of charge–charge interactions with the negatively charged phosphates of the lipid bilayer, thereby creating a small pocket for the CQC motif (Fig 2B). This preferred small pocket orientation, combined with its proximity to the lipid bilayer, supported the notion that the CQC motif Cys residues have reduced conformational freedom, predisposing them to form disulfide bonds more efficiently with limited entropic penalty.

### Binding Studies with the MUC1 CQC Motif

**Fig 3:**
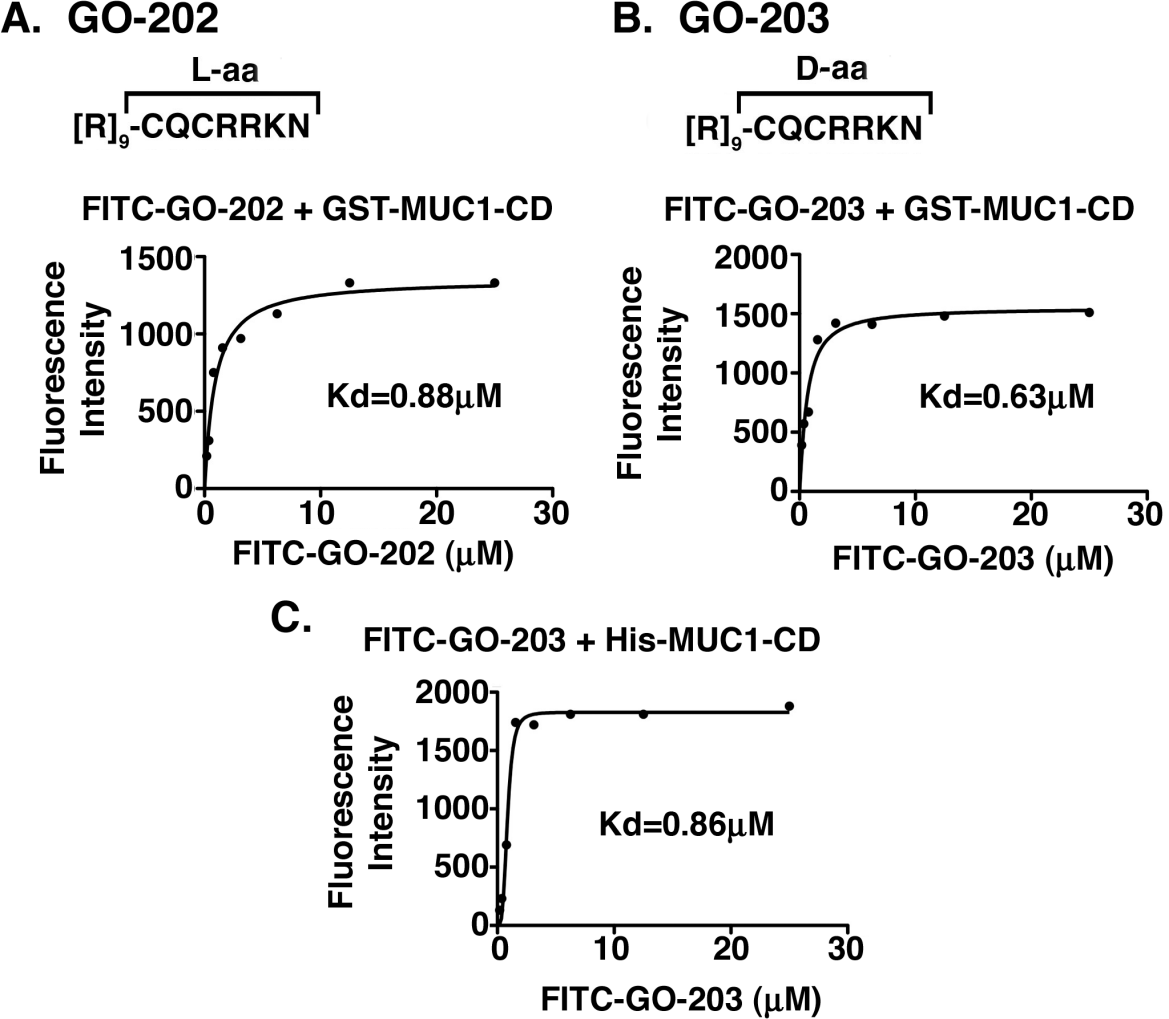
GO-202 and GO-203 bind to the CQC motif. (A) Shown are the L-aa sequence of GO-202 and (B) D-aa sequence of GO-203. The apparent Kd for binding of (A) FITC-GO-202 to GST-MUC1-CD, (B) FITC-GO-203 to GST-MUC1-CD and (C) FITC-GO-203 to His-MUC1-CD proteins were determined by saturation binding experiments.

The MUC1-C CQC motif confers the formation of MUC1-C homodimers necessary for MUC1-C function [7,8]. We therefore generated peptides containing the CQCRRKN sequence as potential agents that could bind to the CQC pocket and block MUC1-C homodimerization. One such peptide, designated GO-202, contains a poly-Arg sequence for cell-penetration linked to CQCRRKN (L-amino acids; Fig 3A). GO-202 was synthesized with an N-terminal FITC label to evaluate direct binding of this peptide to GST-MUC1-CD bound to glutathione-coated ELISA plates. As determined by the saturation binding method, the dissociation constant (Kd) was 0.88 μM (Fig 3A). We also assessed binding of a peptide, designated GO-203, in which the poly-Arg cell-penetrating domain is linked to CQCRRKN (D-amino acids; Fig 3B). Incubation of FITC-labeled GO-203 to GST-MUC1-CD demonstrated a Kd of 0.63 μM (Fig 3B). To confirm specificity of binding, FITC-GO-203 was incubated with His-MUC1-CD bound to nickel-coated ELISA plates (Fig 3C). In these studies, the Kd was 0.86 μM, indicating that the interaction between GO-203 and MUC1-CD is not conferred by the GST- or His-tag. To confirm specificity of the interaction, FITC-GO-203 was also incubated with GST-MUC1-CD in which the CQC motif was mutated to AQA. Here, no interaction was evident between FITC-GO-203 and GST-MUC1-CD(AQA) as supported by detection of only background fluorescence (data not shown). These results demonstrate that both the L- and D-forms of the CQCRRKN peptide directly bind to MUC1-CD and that binding is specific to the CQC motif.

### GO-203 Forms Disulfide Linkages with MUC1-CD at the CQC Motif

**Fig 4:**
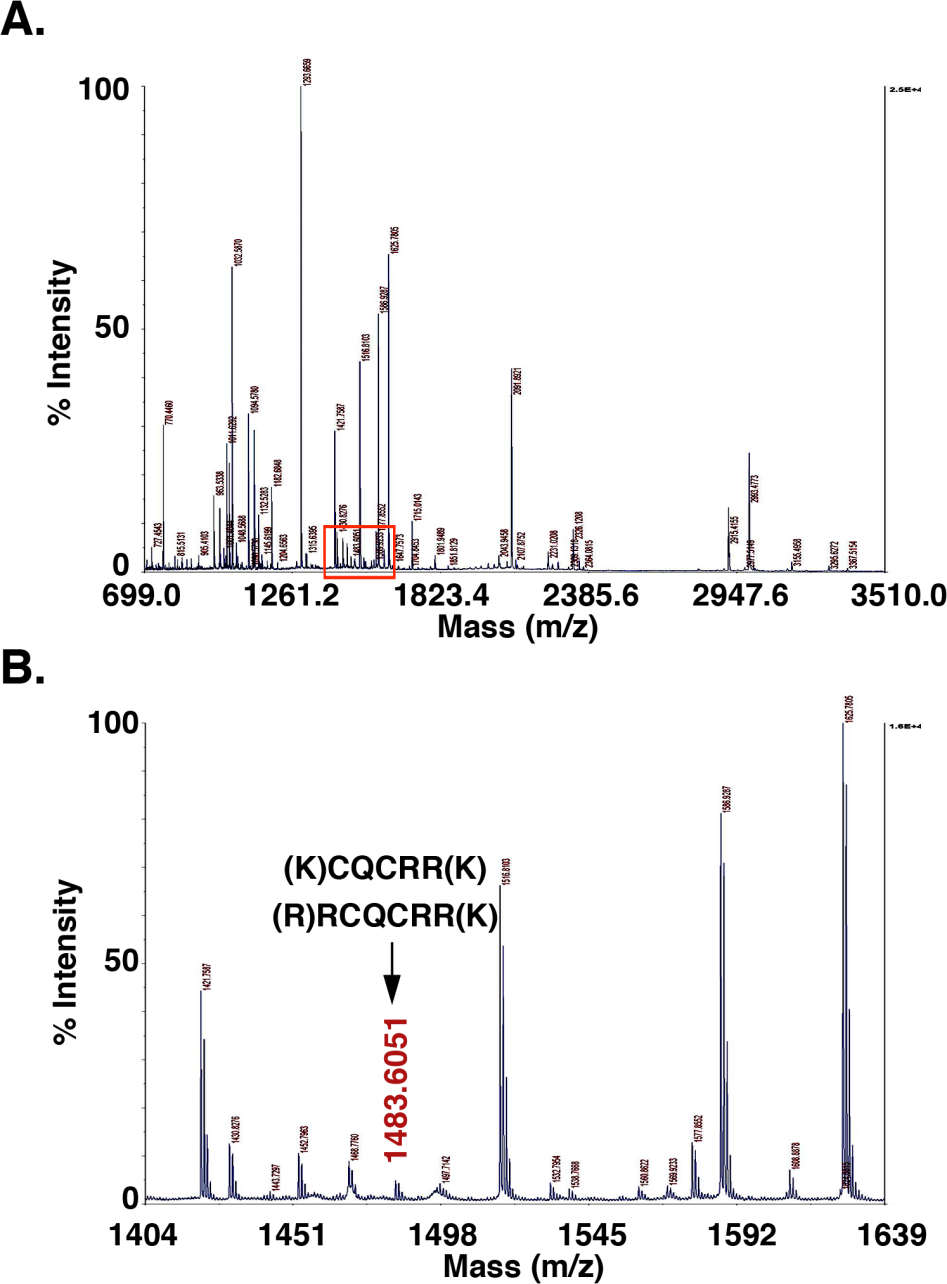
GO-203 forms disulfide linkages with MUC1-CD. (A) Trypsin digest of the 35 kDa GST-MUC1-CD and GO-203 complex was analyzed by MALDI-TOF mass fingerprinting. (B) The highlighted area in (A) was expanded to show a specific peptide peak at an m/z 1483 Da due to the formation of a disulfide bridge between GO-203 and MUC1-CD.

To further assess the association of GO-203 with MUC1-CD, GO-203 was incubated with purified GST-MUC1-CD. The complexes were separated by SDS-PAGE and tryptic digests were analyzed by MALDI-TOF-MS (Fig 4A). Peptide identification was based on MS-bridge analysis of the tryptic digest MALDI-TOF data against the sequences of the 2 known components of the reaction, i.e. GST-MUC1-CD (S1 Fig) and GO-203. The peptide dimer with a single disulfide bridge at the CQC motif was represented by the peak of m/z 1483.6084 Da (Fig 4B) and the properties listed in Table 1. This peak was not observed in the tryptic digests of GST+GO-203 and GST-MUC1-CD samples, indicating that GO-203 forms disulfide bridges specifically with MUC1-CD, and not with GST.

**Table 1:** MS-bridge analysis of MALDI-TOF peptides.

m/z Submitted	MH^+^ Matched	Intensity	Delta ppm	Peptide Combination	Start	End	Missed Cleavage	Sequence
1483.6051	1483.6722	100.0	-45.2	1* 2**	219 9	223 14	1 2	(K)CQCRR(K) (R)RCQCRR(K)

* GST-MUC1-CD

** GO-203

### GO-203 Blocks MUC1-CD Homodimerization *In Vitro*

**Fig 5:**
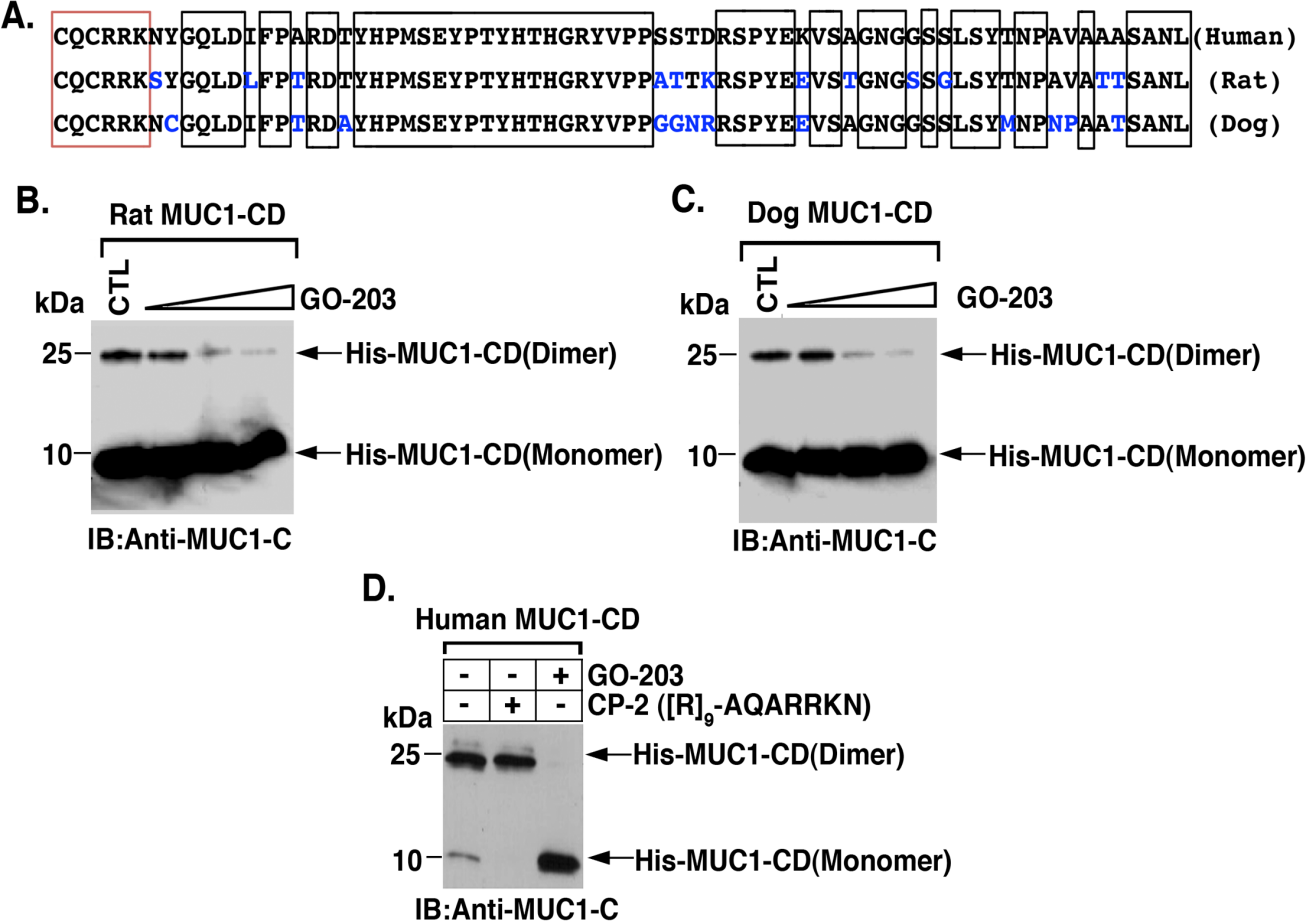
GO-203 blocks MUC1-CD homodimers *in vitro*. (A) Human, rat and dog MUC1-CD proteins share high sequence homology as aligned using Clustal Omega. The conserved regions are shown in the boxes, with the red box denoting the CQCRRK region. (B) Rat and (C) dog purified His-MUC1-CD proteins were incubated with PBS (control) or increasing amounts of GO-203 (50, 150 and 450 μM) for 1 h at room temperature. The proteins were separated in a non-reducing polyacrylamide gel and analyzed by immunoblotting with anti-MUC1-C. (D) Purified human His-MUC1-CD protein was incubated with PBS (control), CP-2 (150 μM) or GO-203 (150 μM) for 1 h at room temperature. The proteins were separated in a non-reducing polyacrylamide gel and analyzed by immunoblotting with anti-MUC1-C.

The MUC1-CD sequence is restricted to mammalian species having appeared late in evolution [6]. The CQCRRK sequence is conserved in the rat, dog and human proteins (Fig 5A). A protein sequence homology analysis using Clustal Omega further demonstrated that human MUC1-CD (AAA60019.1) shares significant sequence homology of 83% with the MUC1-CD proteins of rat (AAI66505) and dog (NP_001181906.1) (Fig 5A). To assess the functional significance of CQCRRK, we generated purified His-tagged rat MUC1-CD protein (~10 kDa) (Fig 5B). Incubation of the rat His-MUC1-CD at pH 7.4 was associated with the formation of ~20 kDa homodimers (Fig 5B). Moreover, addition of GO-203 at increasing amounts relative to His-MUC1-CD decreased homodimerization (Fig 5B). Dog and human His-tagged MUC1-CD proteins also formed homodimers that were effectively inhibited by GO-203 (Fig 5C and 5D). By contrast, the CP-2 peptide, which includes AQARRKN, had no effect on human MUC1-CD homodimerization (Fig 5D).

### GO-203 Blocks MUC1-CD Homodimerization in Cells

**Fig 6:**
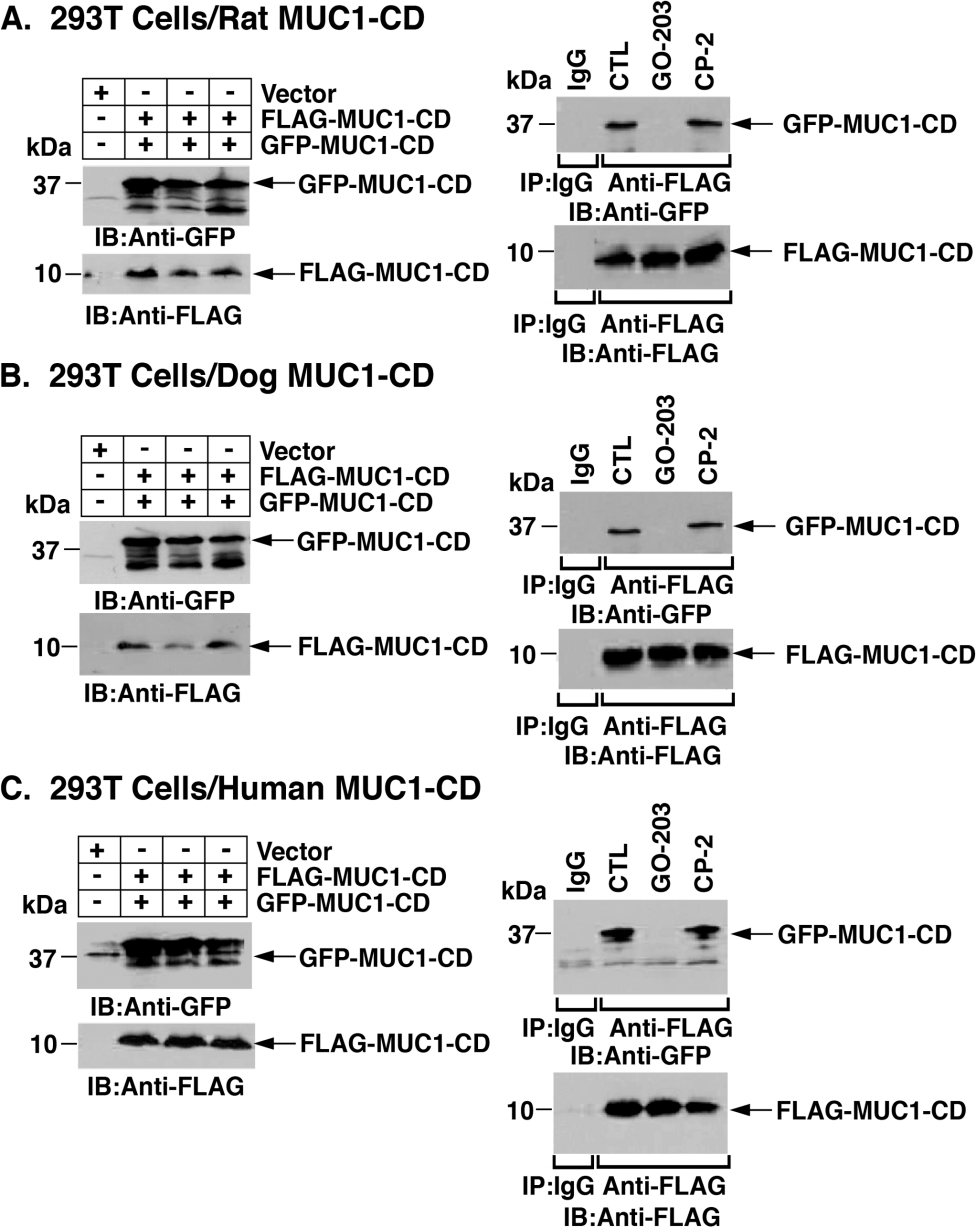
GO-203 blocks MUC1-CD homodimerization in cells. 293T cells were transiently transfected to express an empty vector and (A) rat GFP-MUC1-CD and FLAG-MUC1-CD, (B) dog GFP-MUC1-CD and FLAG-MUC1-CD or (C) human GFP-MUC1-CD and FLAG-MUC1-CD. At 48 h, cells were left untreated (control) or treated with 5 μM GO-203 or CP-2 each day for 3 days. The cells were then harvested for immunoblotting with anti-GFP and anti-FLAG (left panels). Whole cell lysates were also precipitated with anti-FLAG and the precipitates were immunoblotted with the indicated antibodies (right panels).

To further assess the effects of targeting the MUC1-CD CQCRRK motif in cells, we transfected 293T cells to express rat GFP-tagged or FLAG-tagged MUC1-CD (Fig 6A, left). Coimmunoprecipitation studies demonstrated that FLAG-MUC1-CD forms dimers with GFP-MUC1-CD (Fig 6A, right). Notably, treatment of the transfected 293T cells with GO-203 was associated with inhibition of the FLAG-MUC1-CD/GFP-MUC1-CD complexes (Fig 6A, right). By contrast, treatment with CP-2 had no apparent effect (Fig 6A, right). Transfection of 293T cells to express tagged dog MUC1-CD (Fig 6B, left and right) or human MUC1-CD (Fig 6C, left and right) confirmed the formation of MUC1-CD homodimers. Moreover, GO-203 treatment resulted in the inhibition of MUC1-CD homodimerization (Fig 6B, right and 6C, right). In addition, the demonstration that CP-2 treatment has no effect on MUC1-CD homodimer formation supported the specificity of targeting the CQC motif (Fig 6B, right and 6C, right).

### Binding of GO-203 to Endogenous MUC1-C in Cells

**Fig 7:**
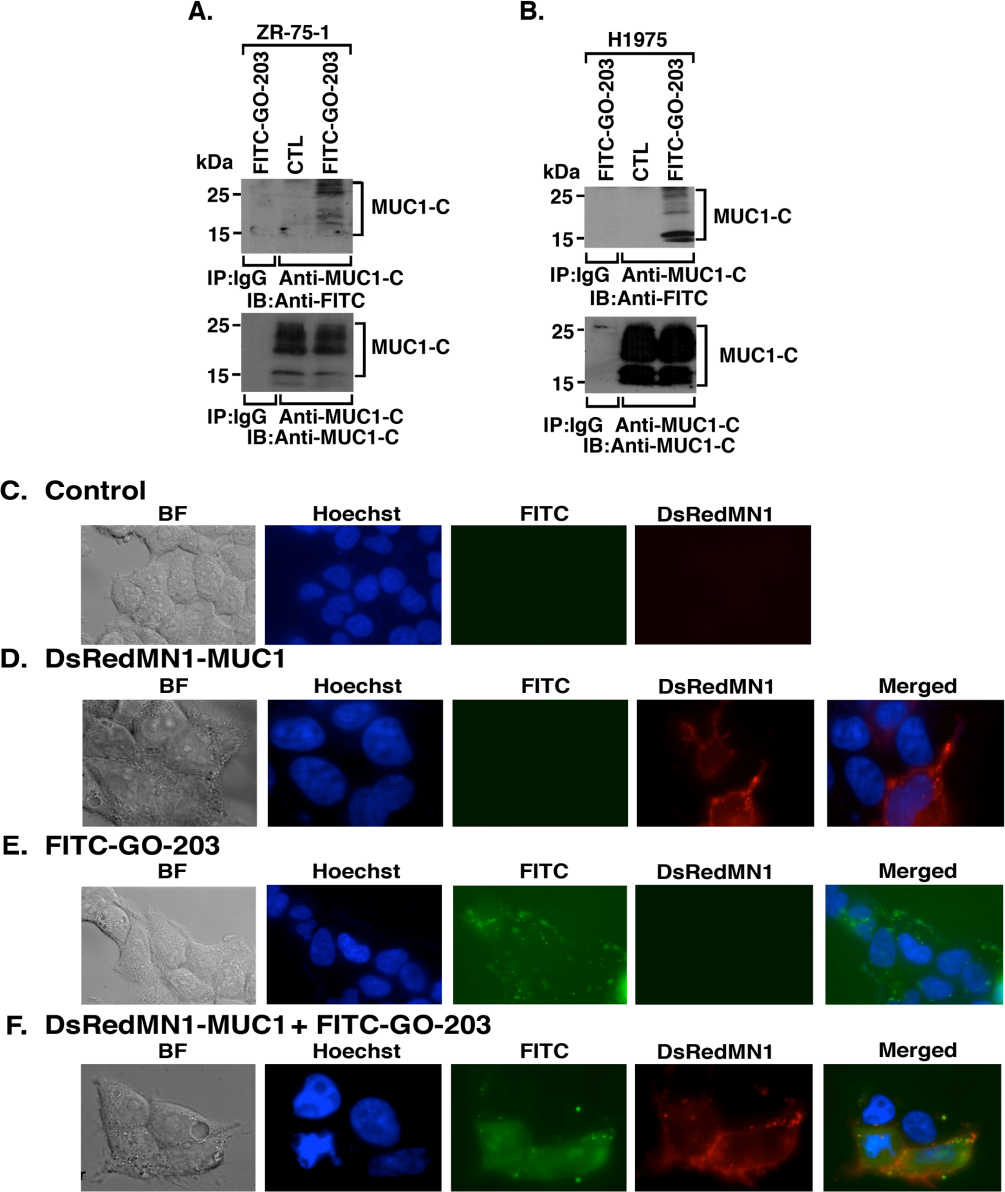
Binding of GO-203 to endogenous MUC1-C. (A) ZR-75-1 and (B) H1975 cells were left untreated (control), and treated with 5 μM FITC-GO-203 overnight. Lysis was performed in a non-reducing buffer. Lysates were precipitated with anti-MUC1-C or a control IgG followed by addition of non-reducing sample buffer. The precipitates were immunoblotted with anti-FITC and anti-MUC1-C. 293T cells were left untreated (C; control), (D) transiently transfected with DsRedMN1-MUC1, (E) treated with 5 μM FITC-GO-203, or (F) transfected with DsRedMN1-MUC1 and treated with 5 μM FITC-GO-203. Distribution of MUC1 (red) or FITC-GO-203 (green) and colocalization (yellow) was assessed by immunofluorescence microscopy. Nuclei were counterstained with Hoechst dye (blue).

To determine if GO-203 associates with endogenous MUC1-C, we treated ZR-75-1 breast cancer cells with FITC-GO-203. Analysis of anti-MUC1-C precipitates by immunoblotting with anti-FITC indicated that GO-203 forms complexes with MUC1-C (Fig 7A). Similar studies performed on FITC-GO-203-treated H1975 lung cancer cells provided further support for the notion that GO-203 associates with endogenous MUC1-C (Fig 7B). MUC1-C is expressed as an ~25–20 kDa N-glycosylated form and as 17/15 kDa unglycosylated species [6,8]. In addition, MUC1-C is detectable as homodimers and higher order oligomers under non-reducing conditions as used in these experiments [8]. In this context and as shown in the entire immunoblots obtained from the results presented above (Fig 7A and 7B), FITC-GO-203 interacts with the different MUC1-C monomeric and oligomeric forms (S2A and S2B Fig). To define the localization of GO-203/MUC1-C complexes, immunofluorescence micoroscopy was performed on 293T cells transfected to express DsRedMN1-labeled MUC1 and/or treated with FITC-GO-203. Control 293T cells incubated with Hoechst dye to stain nuclei had no detectable DsREd or FITC signals (Fig 7C). By contrast, in cells transfected to express DSRedMN1-MUC1, the DSRed signals were predominantly localized along the cell membrane with a few dots detectable in the nucleus (Fig 7D). In 293T cells treated with FITC-GO-203 alone, the FITC signals were localized along the membrane and in the cytosol (Fig 7E). Moreover, in 293T cells expressing DSRedMN1-MUC1 and treated with FITC-GO-203, co-localization of the DSRed and FITC signals (Red+Green→Yellow) was observed along the membrane (Fig 7F). The brighter FITC-GO-203 staining in 293T cells expressing MUC1 as compared to that in the MUC1-null setting could be explained by the retention of FITC-GO-203 in complexes with MUC1-C at the cell membrane.

## Discussion

The MUC1-C transmembrane subunit interacts with RTKs, such as EGFR, at the cell surface and contributes to their activation and downstream intracellular signaling pathways [6,19,23]. Accordingly, there has been emerging interest in the function of the MUC1-C cytoplasmic domain as an oncoprotein. In this context, overexpression of the MUC1-C cytoplasmic domain is sufficient to confer anchorage-independent growth and tumorigenicity [5]. How the MUC1-C cytoplasmic domain induces transformation has been the focus of considerable study; however, little is known about the structure of this oncogenic protein. The present studies thus investigated the physical characteristics of the 72-amino acid cytoplastic domain and demonstrate that, other than the CQCRRK sequence residing adjacent to the cell membrane, the remainder of this protein has no apparent structure.

The absence of identifiable alpha helices or beta-sheets was of interest in that the MUC1-C cytoplasmic domain sequences downstream of the CQC motif are subject to phosphorylation by multiple kinases that include, among others, EGFR, MET, SRC, ABL, GSK3 and PKC [1,4]. In turn, these post-translational modifications regulate interactions of the MUC1-C cytoplasmic domain with effectors of diverse signaling pathways that are associated with transformation [1,4]. As one example, phosphorylation of the MUC1-C cytoplasmic domain confers binding to the WNT pathway effector, β-catenin, and thereby the activation of WNT target genes [5,24]. Other work has linked the MUC1-C cytoplasmic domain to activation of the NF-κB p65 [25,26] and STAT1/3 [27,28] pathways. In this way, the intrinsically disordered structure of the MUC1-C cytoplasmic domain apparently has the capacity to undergo diverse post-translational modifications and to interact with multiple effectors.

Proteins with intrinsically disordered structures are found throughout the genome and include oncoproteins, such as p53 [29], PTEN [30], the androgen receptor [31] and others [14]. These proteins can present challenges for drug development given the absence of pockets or well-defined three-dimensional folds [14]. Therefore, we focused on the design of agents that target the CQC motif, based on the observations that endogenous MUC1-C forms homodimers and the CQC cysteines are necessary and sufficient for this interaction [7,8]. Moreover, we found that mutating the CQC motif to AQA abrogated the capacity of MUC1-C to promote transformation [7]. The present results demonstrate that an L-amino acid [R]_9_-CQCRRKN-containing peptide (GO-202) binds directly to the MUC1-C cytoplasmic domain. Interestingly, similar results were obtained with a D-amino acid [R]_9_-CQCRRKN peptide (GO-203), consistent with a retro-inverso effect in which the CQC motif is flanked on both sides by basic amino acids. Specificity of these cysteine-mediated interactions was confirmed in studies demonstrating that mutating CQC to AQA in the MUC1-C cytoplasmic domain or in the CP-2 control peptide abrogated binding.

In concert with direct binding to the MUC1-C cytoplasmic domain CQC motif, GO-203 was effective in disrupting MUC1-CD homodimers in vitro. In addition, GO-203 treatment inhibited the formation of GFP-MUC1-CD/FLAG-MUC1-CD homodimers in cells, consistent with intracellular binding of GO-203 to tagged-MUC1-CD monomers and thereby blocking availability of the MUC1-C cytoplasmic domain CQC motif for homodimer formation. In this regard and importantly, coimmunoprecipitation studies further demonstrated that FITC-GO-203 associates with endogenous MUC1-C in breast and lung cancer cells. Moreover, confocal studies indicated that GO-203 binds predominantly to MUC1-C at the cell membrane. These findings collectively support the notion that GO-203 binds to the MUC1-C cytoplasmic domain CQC motif in cells and thereby blocks MUC1-C homodimerization and function. Importantly, the present data do not exclude the possibility that GO-203 interacts with other cysteine-containing proteins and therefore is a selective, but not specific, MUC1-C inhibitor.

Based on the above findings and others demonstrating that GO-203, but not CP-2, inhibits the survival of MUC1-C-expressing carcinoma and leukemia cells [19,32], clinical evaluation of GO-203 as a potential therapeutic agent has been underway and is presently being studied in a Phase Ib/II trial for patients with relapsed/refractory leukemia.

## Supporting Information

S1 Fig Amino acid sequence of GST-MUC1-CD. (TIF)

S2 Fig GO-203 forms complexes with MUC1-C monomers and oligomers. (A) ZR-75-1 and (B) H1975 cells were left untreated (control), and treated with 5 μM FITC-GO-203 overnight. Lysis was performed in a non-reducing buffer. Lysates were precipitated with anti-MUC1-C or a control IgG followed by addition of non-reducing sample buffer. The precipitates were immunoblotted with anti-FITC and anti-MUC1-C. Shown are the entire immunoblots from those presented in Fig 7A and 7B. Higher order MUC1-C oligomers are highlighted with an asterisk (*).
(TIF)
