# Supplementary figures and images for: Characterization of the MUC1-C Cytoplasmic Domain as a Cancer Target
Source: PLoS One. 2015 Aug 12;10(8):e0135156. doi: 10.1371/journal.pone.0135156 (PMC4534190; doi:10.1371/journal.pone.0135156)

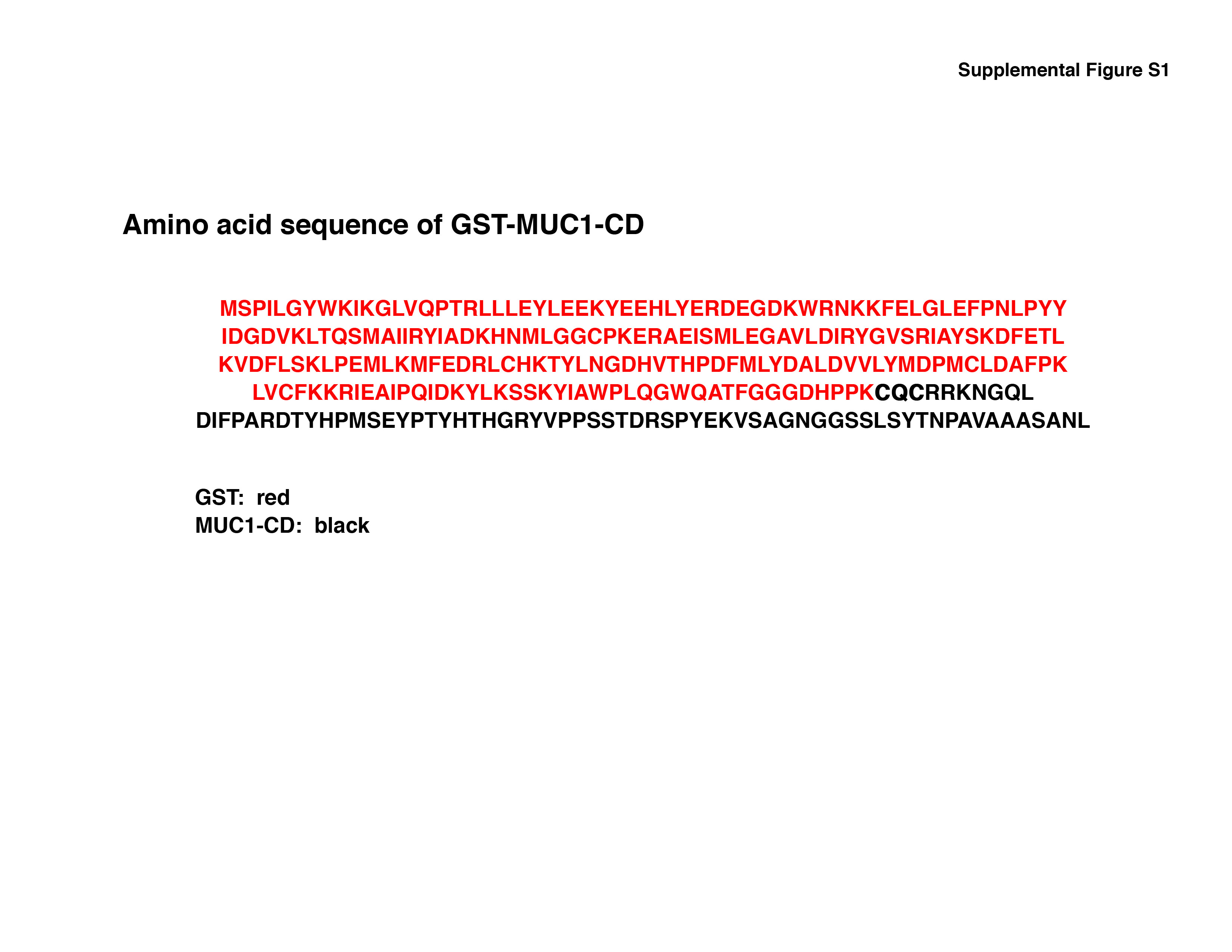

Supplement: S1 Fig — (TIF) [file pone.0135156.s001.tif]

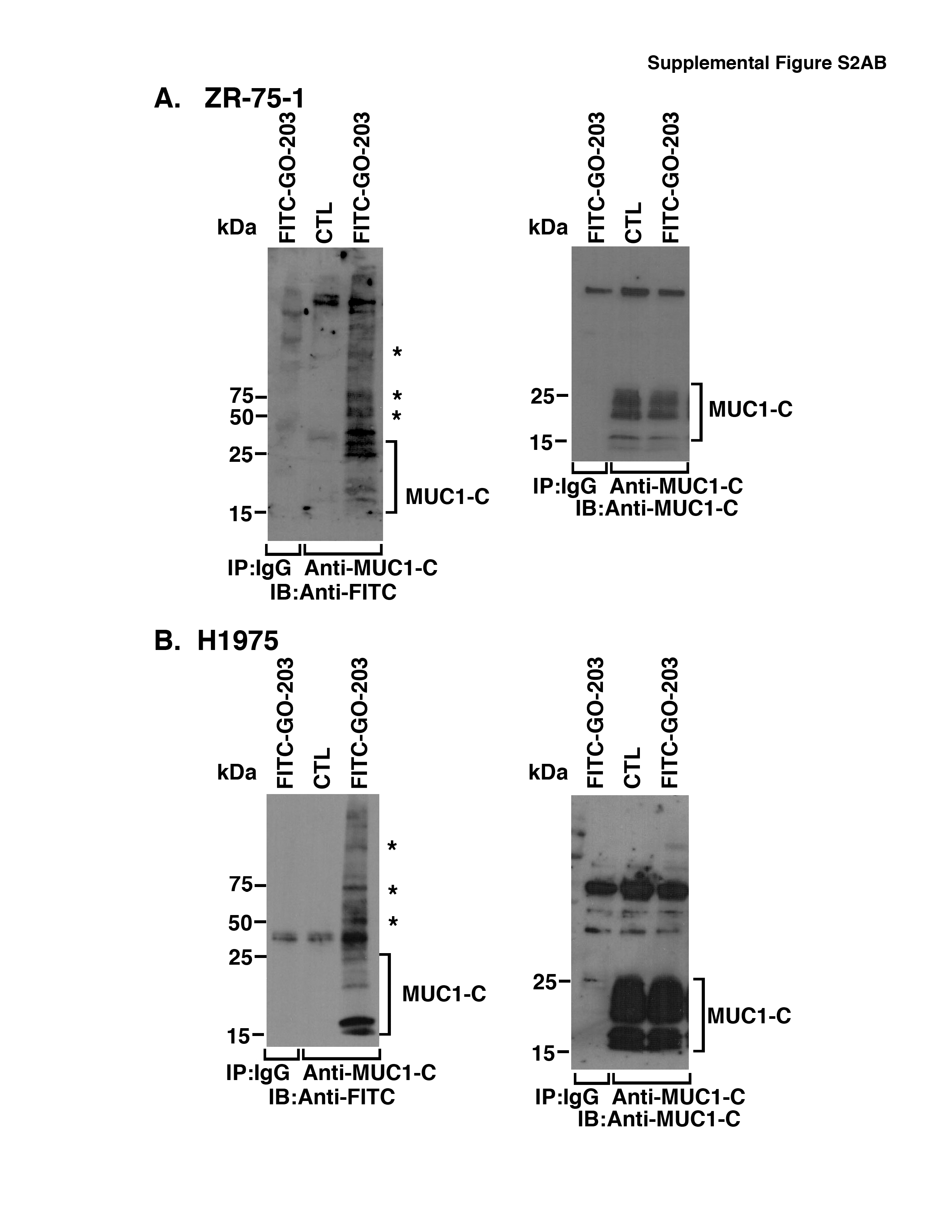

Supplement: S2 Fig — (A) ZR-75-1 and (B) H1975 cells were left untreated (control), and treated with 5 μM FITC-GO-203 overnight. Lysis was performed in a non-reducing buffer. Lysates were precipitated with anti-MUC1-C or a control IgG followed by addition of non-reducing sample buffer. The precipitates were immunoblotted with anti-FITC and anti-MUC1-C. Shown are the entire immunoblots from those presented in Fig 7A and 7B. Higher order MUC1-C oligomers are highlighted with an asterisk (*). (TIF) [file pone.0135156.s002.tif]
